# Chemical Vapor Deposition of Highly Conjugated, Transparent Boron Carbon Nitride Thin Films

**DOI:** 10.1002/advs.202101602

**Published:** 2021-07-03

**Authors:** Paolo Giusto, Daniel Cruz, Tobias Heil, Nadezda Tarakina, Maddalena Patrini, Markus Antonietti

**Affiliations:** ^1^ Max Planck Institute of Colloids and Interfaces Department of Colloid Chemistry Am Mühlenberg 1 Potsdam 14476 Germany; ^2^ Department of Physics University of Pavia Via Bassi, 6 Pavia 27100 Italy

**Keywords:** boron carbon nitride, chemical vapor deposition, optical properties, refractive index, thin films

## Abstract

Ternary materials made up only from the lightweight elements boron, carbon, and nitrogen are very attractive due to their tunable properties that can be obtained by changing the relative elemental composition. However, most of the times, the synthesis requires to use up to three different precursor and very high temperatures for the synthesis. Moreover, the low reciprocal solubility of boron nitride and graphene often leads to BN‐C composite materials due to phase segregation. Herein, an innovative method is presented to prepare BCN thin films by chemical vapor deposition from a single source precursor, melamine diborate. The deposition occurs homogenously at relatively low temperatures generating very high degree of sp^2^ conjugation. The as‐prepared thin films possess high transparency and refractive index values in the visible range that are of interest for reflective mirrors and lenses. Furthermore, they are wide‐bandgap semiconductor with very positive valence band, making these materials very stable against oxidation of interest as protective coating and charge transport layer for solar cells. The simple chemical vapor deposition method that relies on commonly available and low‐hazard precursor can open the way for application of BCN thin films in optics, optoelectronics, and beyond.

## Graphical abstract

Boron carbon nitride thin films recently have attracted much interest due to the wide range of application and tunability of these materials. Here, an innovative method is presented to obtain highly conjugated, visible, transparent BCN thin films via chemical vapor deposition. The prepared films show a high refractive index and high oxidation potential.

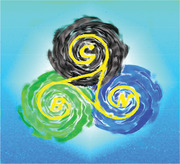

## 1 Introduction

Carbon‐based materials are blossoming, and fullerenes, nanotubes, graphenes, or nanodiamonds indeed expanded science by building units with remarkable properties. The introduction of lightweight neighboring heteroatoms, such as boron and nitrogen, into the pool of sp^2^‐bonded graphitic carbon expands the spectrum of possibilities and is one of the most feasible methods to tailor the electronic and optical properties of carbon materials.^[^ ^1^, ^2^ ^]^ The introduction of structural nitrogen leads to enhanced electrical conductivity (“doping”) and increased oxidation stability of carbon materials.^[^ ^2^ ^]^ On the other hand, the substitution of carbon atoms with boron leads to widening of the bandgap, and the resulting materials have shown p‐type conductivity with increased electron affinity.^[^ ^3^ ^]^ The simultaneous introduction of boron and nitrogen atoms into a carbon structure opens a plethora of possibilities of ternary compositions with their corresponding properties. On the extreme end of high boron and nitrogen content, hexagonal boron nitride BN is the most stable species, a wide bandgap electrical insulator isomorphous to graphene. “Mixing” now graphene and BN, two seemingly very different materials, but with similar lattice parameter and structure, we expect very different properties according to relative composition and atomic arrangements in the lattice.^[^ ^1^, ^4^ ^]^ However, despite the high interest and effort, the synthesis of BCN materials is still challenging, as in most cases carbon‐boron nitride microphase segregation is found, due to reciprocal low solubility, confirmed also by theoretical calculations.^[^ ^1^, ^4^, ^5^ ^]^ On top, most chemical pathways generate a high amount of defects and low *π*‐conjugation along the sheets and require, in most cases, very high synthesis temperatures of 700 °C and above, which vice versa promotes phase demixing and exclude potential low‐temperature homogenous phases to form.^[^ ^2^, ^6^ ^]^

The synthesis of ternary boron carbon nitride (BCN) thin films has been previously addressed by different methods, such as magnetron sputtering, pulsed laser deposition, ion‐beam‐assisted deposition, and chemical vapor deposition (CVD) methods.^[^ ^1^, ^4^, ^6^, ^7^ ^]^ Recently, Ci et al. used a catalytic CVD method on copper substrates from methane and ammonia‐borane precursors.^[^ ^1^ ^]^ They also showed that the method leads to phase segregation of graphene and boron nitride domains within the graphitic layer, i.e., a graphene‐boron nitride superlattice.^[^ ^1^ ^]^ However, the use of single source precursors attracted recently attention due to an improved control of the final materials properties and composition.^[^ ^7^ ^]^ In this regards, alkylamino boranes and alkylborazines have been widely employed for the synthesis of BCN films usually at relatively high temperatures, between 700 and 1300 °C.^[^ ^7^ ^]^ In particular, Beniwal et al. synthesized a boron, carbon, and nitrogen single source precursor, named bis‐BN cyclohexane, to grow ternary graphitic hexagonal BCN monolayers on iridium surfaces at 980 °C.^[^ ^6^ ^]^ Theoretical modeling and experimental evidences suggested that the material obtained in this condition is constituted of B_2_C_2_N_2_, with B_3_N_3_ rings within a graphitic sheet, and homogenous stoichiometry. BCN materials have been exploited recently for their high stability and suitable and tunable band position for (photo‐)redox reactions.^[^ ^8^, ^9^ ^]^ Recently, Wang et al. developed a hexagonal BCN material for hydrogen evolution and CO_2_ reduction under visible light irradiation without the presence of noble metals, by using a ternary mixture of boron oxide glucose and urea at 1250 °C.^[^ ^8^ ^]^ Our group reported also on BCN thin films obtained by a simple test tube method without chemical segregation between carbon–boron nitride phases and tunable optical properties.^[^ ^10^ ^]^

Herein, we present the synthesis of optically transparent BCN thin films with very high refractive index in the visible range from a solid single source precursor, here in detail controlled within a CVD set‐up. The solid precursor, i.e., a melamine‐boric acid adduct, can be considered as nonhazardous and easily available. Although the choice of boric acid, which is set‐up by strong B─O bond, is at a first glance counterintuitive, it will be shown that the B─O bonds are cleaved to form B─N and B─C bonds, driven by aromatization at elevated temperatures. The crystalline melamine‐boric acid adduct can be prepared with different melamine to boric acid molar ratios, here 1 to 1 and 1 to 2, in order to control composition of the final products and to evaluate the different chemical, structural, and optical properties of the resulting BCN thin films, hereafter referred to as BCN‐11 and BCN‐12, respectively. The precursors form a homogenous supramolecular crystal, and its structure was previously determined by Kawasaki et al. to be monoclinic with *P2_1_/c* space group, organized in layers due to hydrogen bonding.^[^ ^11^ ^]^

## 2 Results and Discussion

The as‐prepared precursors were analyzed by Fourier‐transform infrared spectroscopy and X‐ray diffraction (Figure S1a,b, Supporting Information) to confirm that the melamine‐boric acid adducts were successfully formed, and the spectra are in good agreement with previous reports.^[^ ^11^, ^12^ ^]^ To evaluate the potential of these single‐source precursors in the deposition of BCN thin films, their decomposition was followed via thermogravimetric analysis (TGA; Figure S2, Supporting Information). As shown by the TGA, both melamine‐boric acid adducts cannot be thermally sublimed, with ≈20% leftover mass detected at 1000 °C. Therefore, the deposition exploits the gaseous by‐products produced by thermal decomposition of the precursor which eventually polymerize on the substrate surface. As previously introduced, the thermal cleavage of the B─O bonds occurs due to the particular supramolecular crystal organization and orientation in the melamine‐boric acid adduct. As shown in TGA‐mass spectrometry (MS) analysis (Figure S3, Supporting Information), the thermal decomposition occurs in two main steps, the lower temperature one below 200 °C and a second one, starting at about 450 °C. The first one occurs with main release of condensed small molecule byproducts, such as NH_3_ (*m*/*z* = 17) and water (*m*/*z* = 18). At higher temperatures, the precursor condenses further, releasing higher masses fragments which were attributed to volatile oxides (CO_2_^+^, NO^+^, NO_2_^+^ *m*/*z* = 44, 30, 46) which eliminate the majority of oxygen, and boron‐, carbon‐, nitrogen‐ and oxygen‐containing fragments (BC^+^/CO_2_^++^, CN^+^/BNH^+^, CNO^+^/BO_2_^+^, CO_2_^+^/OCNH_2_^+^, and B_2_C_2_^+^/CO_2_H^+^ at *m*/*z* = 22, 26 42, 43, 44, and 45, respectively). As previously reported, melamine de‐oligomerizes slightly below 400 °C, which apparently sets in the further decomposition cascade via cyanamide reactions.^[^ ^13^ ^]^

The evolved gaseous fragments from the precursor suggest that melamine‐boric acid is a convenient precursor for effective deposition of ternary BCN thin films, and their evaluation give insights on the mechanism of the gaseous phase fundamental for CVD deposition. The low temperature evolution of ammonia, water, and in the second stage of NO*_x_*, points to the formation of B─C bonds, which as shown in the following, will be kept into the ternary BCN thin films. Furthermore, the thermal decomposition profile allows for defining the temperature to be used for CVD deposition: a first lower temperature for the precursor evaporation is set at 500 °C, and a second higher one at 550 °C, for the gaseous precursor polymerization on the substrate surface. We call this process subtractive‐reactive‐CVD, as the vapors are “subtracted” from the solid precursor eventually react or re‐polymerize on the substrate surface, at higher temperature and in a different structure. The ternary BCN thin films deposited this way are highly homogenous and with negligible surface roughness, as observed by naked eye from their mirror‐like surface reflectivity, but also quantified by scanning electron microscope (SEM) and atomic force microscope (AFM) characterizations (Figure S4, Supporting Information), with a slightly higher evidence of formation of valleys for BCN‐12 attributed to the presence of crystalline features, as shown in the following. The high homogeneity and flatness are of paramount importance for the application of BCN thin films in optics, photonics, and beyond.

**Figure 1:**
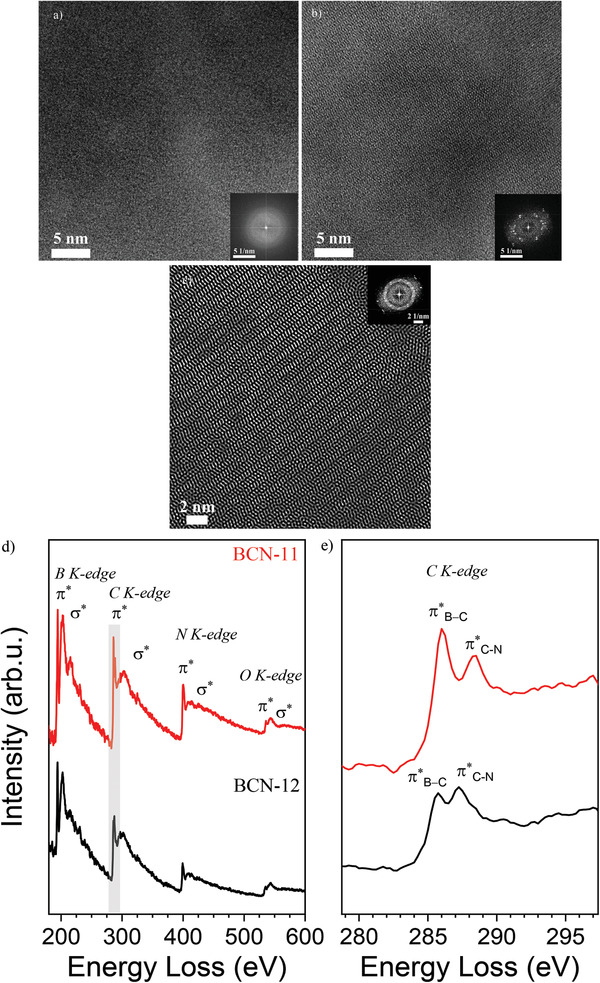
TEM images of a) BCN‐11 and b,c) BCN‐12 with corresponding fast Fourier transformations in insets; d) EELS spectra showing the typical energy loss range of boron, carbon, and nitrogen elements; e) zoom of (d) in the range 279–297 eV (red line, BCN‐11; black line BCN‐12).

At the nanoscale, the ternary thin films show very different morphologies between BCN‐11 (**Figure** **1**a) and BCN‐12 (Figure 1b). On the one hand, BCN‐11 thin films show an apparently amorphous structure (Figure 1a). On the other hand, BCN‐12 films present an organized, semi‐crystalline structure (Figure 1b,c), as confirmed by the fast Fourier transform (FFT; inset in Figure 1b). The FFT of the BCN‐12 sample shows a pattern, usually attributed to the overlap of a few (two or three) crystalline domains. We could not index obtained patterns in any known structure of the B‐C‐N system. Looking at single sites where the film is sufficiently thin, we observe typical herringbone structures, with the graphitic stacking of ≈1 nm sized graphenes zig‐zagged through the structure. At this level, it however must stay unclear if this is a consequence of epitaxy or the real structure. A detailed structural investigation will be the aim of a following work.

In order to prove the thin films contain boron, carbon, and nitrogen, all involved in a joint electronic conjugated system, the samples were further analyzed by electron energy loss spectroscopy (EELS; Figure 1d) recorded at the same, previously shown transmission electron microscope (TEM) image areas. The spectral bands of each element are very well resolved and show a sharp and intense peak corresponding to 1s‐π* antibonding orbital, followed by a wider 1s‐σ* antibonding orbital. This is typical for highly extended, delocalized *π*‐electron systems.^[^ ^1^, ^2^ ^]^ The spectra thereby reflect a very well‐defined structure with very long sp^2^‐hybridized structure, including all boron, carbon, and nitrogen elements.^[^ ^2^ ^]^ The EELS spectra of the BCN‐11‐BCN‐12 samples show the three K‐edges at about 194.25–194.5, 285.75–286, and 399.25–400.5 eV, with only minor differences between the two samples, corresponding to characteristic K‐shell ionization edges of boron, carbon, and nitrogen, respectively. Moreover, that the C 1s‐π* antibonding orbital reveals a double sharp peak, at about 286 and 288 eV, revealing two different state of oxidation for the element, although both strongly conjugated (Figure 1d).^[^ ^8^ ^]^ This suggests that carbon creates conjugated bonds both with boron, being in a slightly more reduced state (286–285.75 eV) and with nitrogen in a more oxidized one (288.5–287.25 eV), due to the higher electronegativity with respect to carbon. Further, the BCN‐12 peaks appear in all cases at lower energy losses, thus suggesting a significantly more positive valence band (more positive work function) with respect to BCN‐11. At higher energy losses, a small contribution of oxygen (at about 535 eV) appears although with low intensity 1s‐π* peak, suggesting the presence of adsorbed water and CO_2_, or minor oxygen defects, e.g., at grain boundaries.

Previous attempts for the synthesis of ternary BCN materials from *N*,*N*′‐ethylmethylimidazolium tetracyanoborate, in spite of the rather high synthesis temperature (usually above 1000 °C), achieved only a lower extent of conjugation when compared to the present work, and allow a direct EELS‐based visualization of the improvements.^[^ ^2^ ^]^ Importantly, in a graphene‐boron nitride superlattice, as reported by Ci et al., the energy loss peaks of boron, carbon, and nitrogen appear at significantly lower energies with respect to the present case (up to 11 eV for carbon), suggesting a very different electronic environment.^[^ ^1^, ^14^ ^]^ The large energy loss shift is attributed to a very different electron density of the elements between the two cases and, therefore, graphene‐boron nitride phase separation can be practically excluded.^[^ ^15^ ^]^ Moreover, the large shift to higher binding energies reveals that our both systems have a very positive highest occupied molecular orbital levels and thus very stable against oxidation.^[^ ^8^, ^16^ ^]^

Further insights on the chemical composition and bonds in BCN thin films were obtained by X‐ray photoelectron spectroscopy (XPS). The relative average chemical composition obtained from XPS spectra of BCN‐11 and BCN‐12 are BC_3.4_N_3.4_ and BC_2.8_N_2.5_, respectively, confirming that in both cases significant amounts of boron are successfully incorporated into the thin film structure. As mentioned previously, the chemical composition is in good agreement with the TGA‐MS analysis: indeed, the precursors possess a relative composition of BC_3_N_6_ (BCN‐11) and B_2_C_3_N_6_ (BCN‐12), and the final thin film is relatively depleted in nitrogen, attributed to the evolution of ammonia below 200 °C, and to the elimination of NO*_x_* above 450 °C.

**Figure 2:**
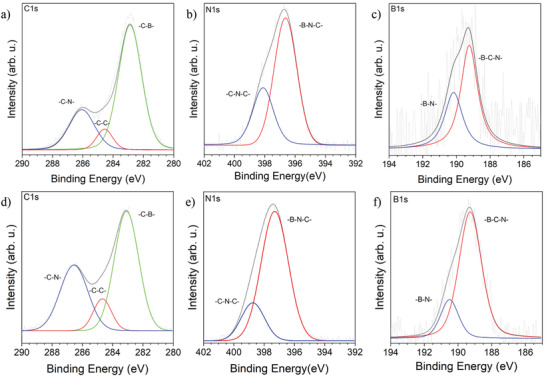
XPS measurements of BCNs thin films. BCN‐11 deconvoluted peaks of a) carbon, b) nitrogen, c) boron. BCN‐12 deconvoluted peaks of d) carbon, e) nitrogen, and f) boron.

The chemical composition is moreover in contrast with phase separation hypothesis: indeed, if for graphene‐boron nitride phase separation we expect almost the same relative atomic content of boron and nitrogen. Here, the relative amount of boron increases slightly for BCN‐12, while the C/N ratio is kept almost constant. This also suggests that the structure is not a graphene with carbon pairs replaced by ─B═N─ units, but rather a CN‐material where boron is introduced into the chemical structure.^[^ ^10^ ^]^ Deconvolution of XPS spectra indeed nicely confirms this assumption, providing insights into the chemical bonding scheme within the two materials. In both cases, the carbon peak is asymmetric, which is indicative of overlapping, different bonding states.^[^ ^17^ ^]^ The deconvolution of the C 1s peaks shows for both samples—very unexpectedly—a strong preference of carbon to create bonds with heteroatoms, as clearly reflected by the intensity of the three peaks at 282.9–283.1 eV C─B, 284.5–284.6 eV sp^2^ C─C, and 286.1–286.7 eV sp^2^ C─N (**Figure** **2**a,d). Again, this confirms the absence of the phase separation.^[^ ^18^, ^19^ ^]^ Furthermore, the N 1s spectra show two components that were attributed to sp^2 ^B─N─C bonding scheme at 396.6 (BCN‐11) and 397.5 (BCN‐12) eV and sp^2^ C─N─C at 398.1 (BCN‐11) and 398.6 (BCN‐12) eV environments within the structure (Figure 2b,e).^[^ ^18^ ^]^ The only significant difference between BCN‐11 and BCN‐12 nitrogen deconvoluted peaks accounts for the more positive work function at nitrogen in BCN‐12.^[^ ^20^ ^]^ Moreover, the B 1s spectrum shows two components centered at 189.2 and 190.2–190.5 eV attributed to sp^2^ B─C─N and B─N environments, due to the lower electronegativity of carbon with respect to nitrogen (Figure 2c,f).^[^ ^1^, ^21^ ^]^ The deconvoluted peaks lie in the typical range of sp^2^ conjugated boron.^[^ ^22^ ^]^

Overall XPS data confirm the presence to highly conjugated ternary BCN materials and are in good agreement with the EELS analysis.^[^ ^1^ ^]^ Of note, the peak usually attributed to B─O bonds, lying in the range 192–192.6 eV, is not present, confirming that despite the high oxophilicity of boron, this CVD method creates B─C and B─N bonds in a conjugated structure at comparably low temperatures.^[^ ^2^, ^18^, ^21^, ^22^ ^]^ This is further confirmed by the deconvolution of the O 1s (Figure S5, Supporting Information) which shows a single peak at 530 eV, and attributed to CO_2_ or H_2_O, where B─O bonds would occur at around 533 eV.^[^ ^22^ ^]^

The high homogeneity and flatness achieved for the BCN thin films allow for applications in optics as transparent and highly refractive coatings. Here, the film quality requirements are strict to avoid, for instance, undesired scattering effects. Both BCN‐11 and BCN‐12 thin films are highly transparent in the visible range as confirmed by transmittance spectra (Figure S6, Supporting Information), the absorption onset occurring in both cases at wavelengths shorter than 450 nm. Increasing the boron content in the melamine‐boric acid solid precursor leads, via the higher boron content in the ternary thin films, to a blue shift of the absorption.^[^ ^10^ ^]^

**Figure 3:**
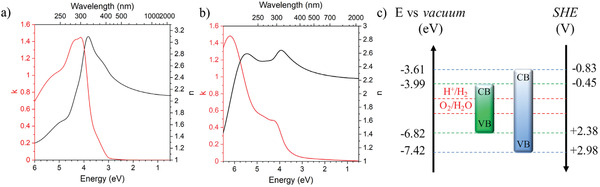
Optical functions of a) BCN‐11 and b) BCN‐12 (in red, extinction coefficient *k*; in black, refractive index *n*). c) Energy levels of BCN‐11 (green) and BCN‐12 (blue); CB is the conduction band, VB the valence band energy levels.

BCN materials are expected to have very high refractive index with high transparency in the visible range.^[^ ^23^ ^]^ Therefore, our BCN thin films were characterized by variable angle spectroscopic ellipsometry (VASE) over a broader spectral range which allows for modeling the dispersion and absorption properties. By combining VASE, reflectance, and transmittance spectra analysis, it is possible to determine the optical functions. The refractive index and extinction coefficient spectra at a first glance reveal a very different behavior of BCN‐11 and BCN‐12 (**Figure** **3**a,b). On the one side, the spectra of BCN‐11 have a single broad peak, both for the extinction coefficient and the refractive index functions, which is typical for disordered materials.^[^ ^24^ ^]^ On the other side, the BCN‐12 spectra show well‐defined peaks, characteristic of crystalline semiconductors.^[^ ^25^ ^]^ Similar spectral shapes were previously reported for germanium and silicon (although at higher energy values), where the peaks in the extinction coefficients were attributed to direct optical transitions.^[^ ^26^ ^]^ The different features of the optical functions are in good agreement with the morphologies depicted in the TEM images (Figure 1a,b). Both BCN thin films absorb strongly in the UV range below *λ* = 400 nm (3.1 eV) with extinction coefficients maxima of 1.45 (at 4.13 eV) and 1.48 (at 6.2 eV) for BCN‐11 and BCN‐12, respectively. The extinction coefficients are very high when compared to organic semiconductors, which values are usually around 1, and in the range of inorganic vis‐transparent semiconductors, such as TiO_2_, where maxima values range from 1.5 to 1.8 for amorphous and anatase thin films, respectively.^[^ ^24^, ^27^ ^]^ The elaboration of the optical properties allows to define also the absorption coefficient spectra (Figure S7, Supporting Information). The latter curves confirm that the light absorption increases and toward the UV range with increasing boron content in the material, up to values of 6.28 × 10^5^ and 9.37 × 10^5^ cm^–1^ at 281 and 198 nm, respectively, for BCN‐11 and BCN‐12. The high absorption coefficient in the UV‐C range makes BCN materials also promising for UV‐protection without compromising color or visible light transmission. The real part of the refractive index (*n*), is very high compared to other organic materials with high transparency in the visible range, but lower than diamond (*n*_D_ = 2.42) or the recently reported carbon nitride films (*n*_D_ = 2.43)^[^ ^28^, ^29^ ^]^ or inorganic materials such as titania with (*n*_D_ ≈ 2.5).^[^ ^28^, ^30^ ^]^ The real part of the refractive index function gives *n*_D_ values of 2.22 and 2.28 for BCN‐11 and BCN‐12, slightly lower than recently reported carbon nitride thin films, which we attribute to the introduction of boron into the covalent structure, lowering the polarizability when compared to the pure graphene or carbon nitride species. The refractive index dispersion versus the wavelength, described here in terms of Abbe number, increases from 3.4 to 26 for BCN‐11 and BCN‐12, respectively, which makes correctly designed BCNs appealing for application as transparent reflective coatings, lenses, and encapsulants for light‐emitting diodes to increase the light‐extraction efficiency.^[^ ^31^, ^32^ ^]^ As a reference, titania has a higher refractive index dispersion as reflected by a lower Abbe number of 9.87.^[^ ^32^ ^]^

Besides the extraordinary optical properties, BCN thin films are of high interest also for their electronic work functions and their possible application for heterojunction‐based devices. The energy levels (Figure 3c) reveal that the bandgap widens with increasing boron content from 2.83 eV for BCN‐11 to 3.81 eV for BCN‐12, as determined by the Tauc plot method from the absorption coefficient spectra (Figure S8, Supporting Information), assuming an indirect bandgap. Increasing the boron content also gives record‐low positive valence band minima of +2.38 and +2.98 V (versus standard hydrogen electrode), as evaluated by ultraviolet photoelectron spectroscopy (UPS, Figure S9, Supporting Information) comparable and even higher than carbon nitrides and most of inorganic oxides such as TiO_2_ (+2.76 V) (Figure 3c).^[^ ^28^, ^33^ ^]^ This provides an unusually high stability to these covalent materials against oxidation.^[^ ^34^ ^]^ Therefore, the as‐prepared BCN coatings are also of high interest as anodes for electrochemistry at high driving voltages, transparent charge transport layers in solar cells, and as protective coatings.^[^ ^33^, ^35^ ^]^

BCN‐11 shows bright fluorescence emission in the blue spectral range (Figure S10, Supporting Information). However, while no photoluminescence could be recorded for BCN‐12. This is presumably due to the presence of trap states at the surface or at grain boundaries which reduces the radiative electron–hole recombination.^[^ ^36^ ^]^

## 3 Conclusion

In summary, an innovative CVD method for depositing ternary BCN thin films based on a simple and economic single‐source precursor was presented. The deposition of BCN occurs at relatively low temperature of 550 °C while granting a high degree of conjugation for all the elements. Increasing the boron content in the precursor successfully increases the boron content while keeping a strongly sp^2^ conjugated environment in the deposited films. This increased boron content leads also to an increased crystallinity and stability of BCNs to oxidation. In terms of optical properties, BCN thin films have shown very high refractive index in the order of 2.2, with features in the optical functions features reflecting the structural order depicted in the TEM investigations. The refractive index function of BCN‐12 shows low optical dispersion, which makes it a good candidate for lenses and to produce highly reflective and transparent coatings. The method presented here shows also the possibility to tune bandgap and energy levels position by increasing the boron content. The valence bands obtained by optical measurements show highly positive values of up to +2.98 V, providing ultra‐noble materials for electrochemistry and for electrode protection. Eventually, besides the extraordinary optical properties the BCN thin films can also find application as anodes in electrochemical processes, as charge transport layer in solar cells, and protective coating against UV and environmental oxidation.^[^ ^35^, ^37^ ^]^

## 4 Experimental Section

### Synthetic Procedure for BCN Thin Films by CVD

The preparation of BCN thin films was done with a planarGROW‐3S‐OS CVD System for Organic Semiconductor, provided by planarTECH, with a 3 in. quartz tube. In a typical recipe, a 2 in. fused silica (1 mm thickness) and a silicon (0.28 mm n‐type silicon (100) with native oxide layer) substrates, were thoroughly rinsed with deionized water, ethanol, isopropanol, and acetone before use, and placed vertically in the center of the second CVD oven. A glass boat contained the BCN‐11 precursor (5 g) (or 7 g BCN‐12 precursor) in the center of the first oven. Then, the vacuum was pulled down to 10 Torr and the reactor was flushed for 10 min with pure nitrogen (500 sccm). The temperature at the substrate was raised to 550 °C in 40 min, with 50 sccm nitrogen flow as carrier gas. Then, the solid precursor was heated up to 500 °C at a rate of 16 °C min^−1^, and kept at this temperature for 120 min. The substrates were kept at 550 °C for 180 min.

### TEM and EELS

The measurements were acquired using a double‐Cs‐corrected Jeol JEMARM200F microscope, equipped with a cold field emission gun, a Gatan GIF Quantum detector, and a JED‐2300 energy‐dispersive X‐ray detector. The acceleration voltage was typically set to 80 kV. Of note, the samples were prepared by directly depositing the BCN films on the carbon side of the TEM grid.

### XPS and UPS

XPS measurements were performed using CISSY equipment with a SPECS XR 50 X‐ray gun Mg K*α* radiation (1254.6 eV) and Combined Lens Analyzer Module (CLAM), on samples deposited on silicon substrates. C, N, and O 1s spectra were collected with five acquisitions of 100 s. B 1s spectra were collected with ten acquisitions of 100 s, to improve the signal while avoiding excessive charging effects. UPS was collected in the same setup equipped with a SPECS UVS 10/35 used in an ultrahigh vacuum chamber (UHV) with He I (21.2 eV) radiation source. The detector was a CombinedLens with an analyzer module thermoVG (TLAM). The Fermi level of gold reference was used to calibrate the system.

### Spectroscopic Ellipsometry Characterization

Spectroscopic ellipsometry measurements were performed in different points by using VASE instrument by J. A. Woollam Co. equipped with rotating analyzer and AutoRetarder in the range 250–2500 nm at different angles of incidence on silicon (65°, 70°, 75°) and fused silica (55°, 60°, 65°) substrates. Transmittance (normal incidence, only for fused silica substrates) and reflectance (8°, both on fused silica and silicon substrates) were measured with Varian Cary 6000i spectrometer in the spectral range 200–1800 nm. Measurements and data modeling by WVASE32 software assumed the samples to be isotropic, deriving the optical function, absorption coefficient, and thickness values. The optical functions derived were obtained as best fit from ellipsometry, reflectance, and transmittance for the BCN samples deposited on silicon and fused silica substrates. From the optical functions depicted in Figure 3a,b, the samples thickness could also be derived on each substrate: 1.3 nm for BCN‐11 and 0.8 nm for BCN‐12 on silicon substrate, 3.6 nm for BCN‐11, and 0.4 nm for BCN‐12 on fused silica substrates.

## Conflict of Interest

The authors declare no conflict of interest.

## Supporting information

Supporting Information

## Data Availability

Research data are not shared.
