# Supplementary material for: Chemical Vapor Deposition of Highly Conjugated, Transparent Boron Carbon Nitride Thin Films
Source: Adv Sci (Weinh). 2021 Jul 3;8(17):2101602. doi: 10.1002/advs.202101602 (PMC8425861; doi:10.1002/advs.202101602)
Supplement: Supplementary file 1 — Supporting Information [file ADVS-8-2101602-s001.pdf]

© 2021 Wiley-VCH GmbH

ADVANCED SCIENCE
Open Access

Supporting Information

for *Adv. Sci.*, DOI: 10.1002/advs.202101602

# Chemical vapor deposition of highly conjugated, transparent boron carbon nitride thin films

*Paolo Giusto,* Daniel Cruz, Tobias Heil, Nadezda Tarakina, Maddalena Patrini, and Markus Antonietti**

# Supporting Information

# Chemical vapor deposition of highly conjugated, transparent boron carbon nitride thin films

*Paolo Giusto, Daniel Cruz, Tobias Heil, Nadezda Tarakina, Maddalena Patrini and Markus Antonietti*

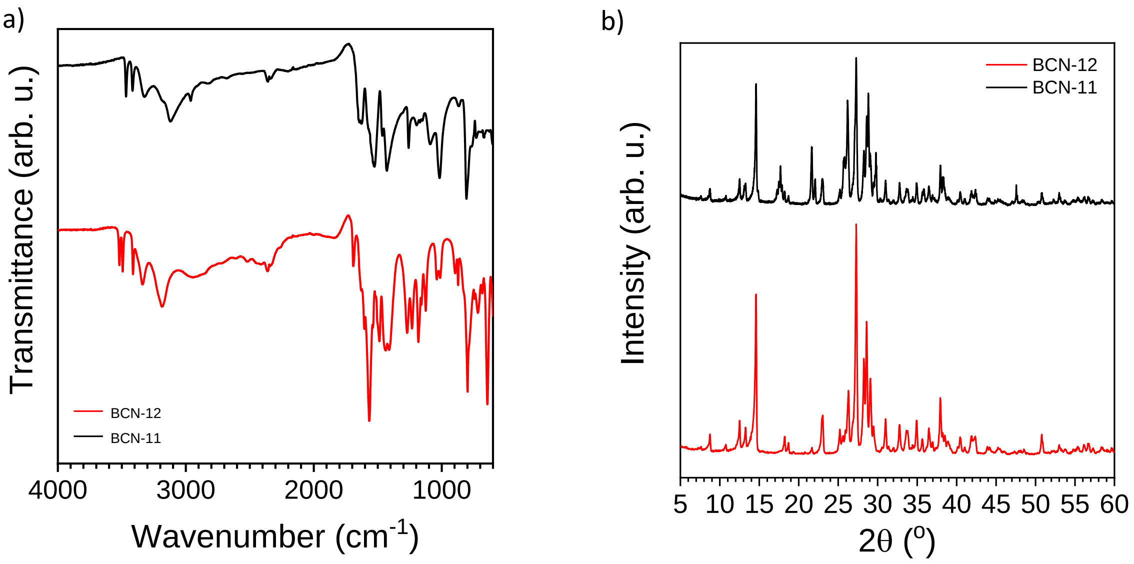


FIG S1. FTIR (a) and XRD (b) of BCN-12 (red line), BCN-11 (black line).

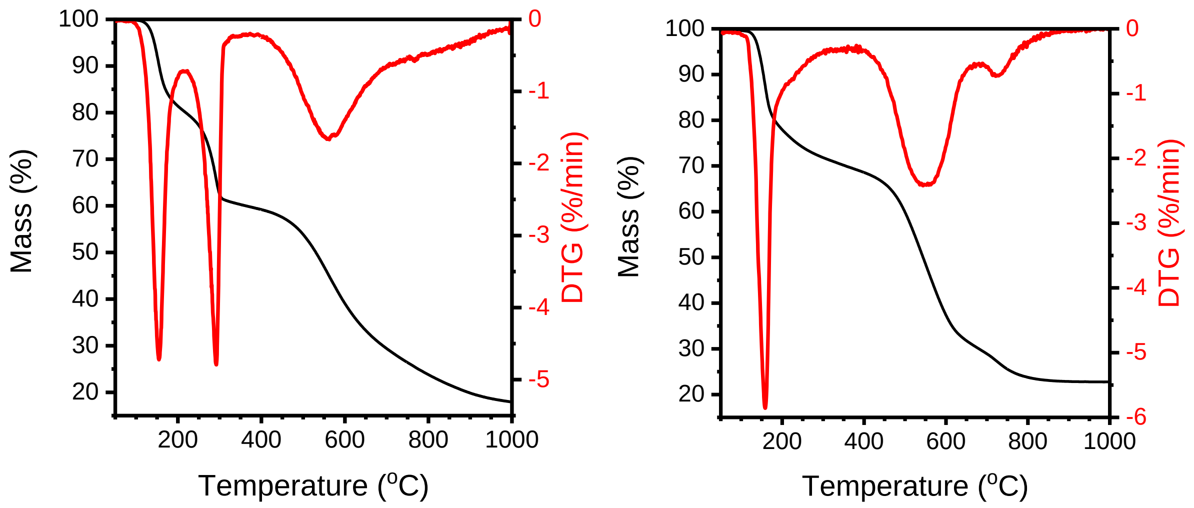


Figure S2. TGA and derivate (DTG) of BCN-11 (left) and BCN-12 (right) precursors.

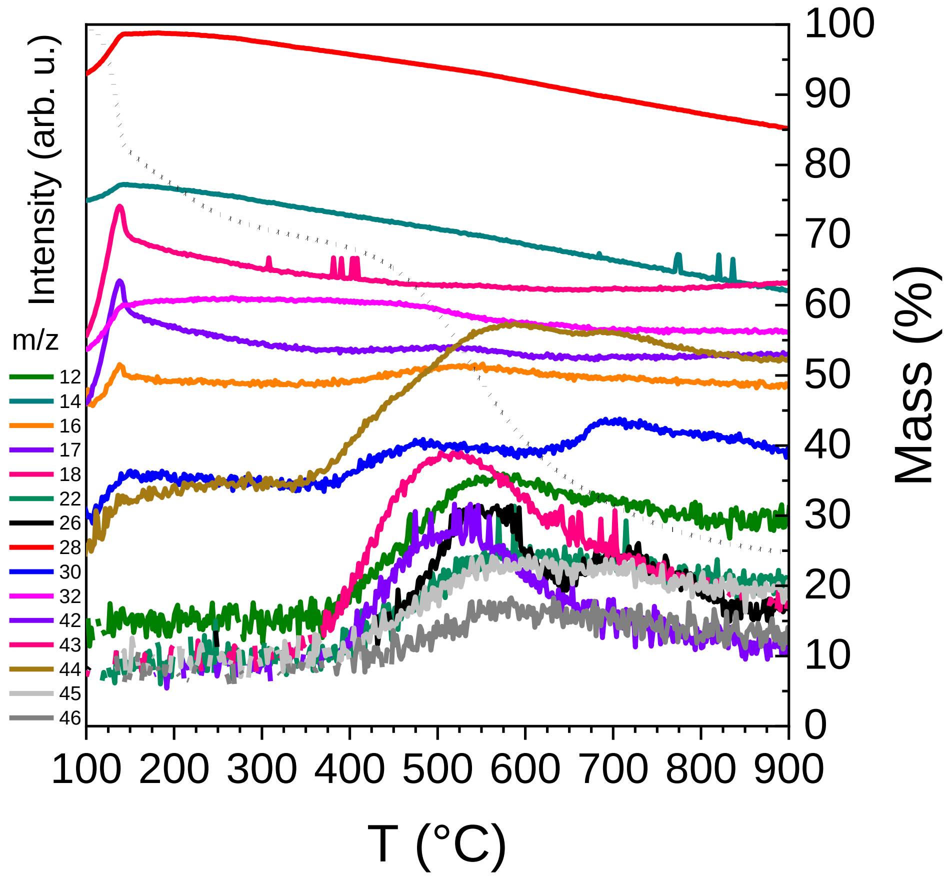


Figure S3. TGA MS of BCN-12 precursor. Dotted line is the TGA, solid lines MS at different m/z.

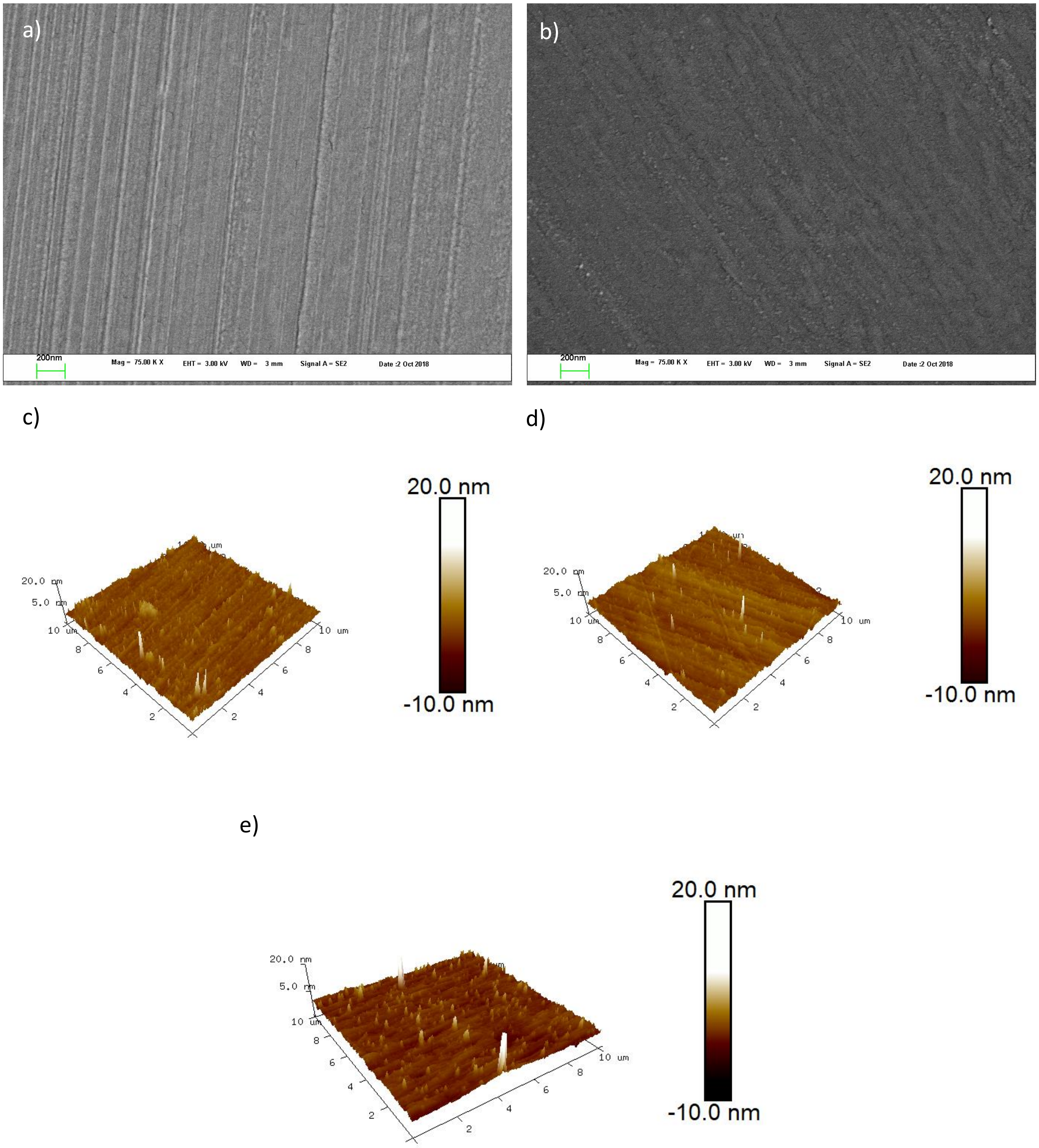


Fig S4. SEM images of BCN-11 (a) and BCN-12 (b). AFM of BCN-11 (c) and BCN-12 (d) on fused silica substrates. (e) AFM of bare fused silica substrate. Roughness (Rq): 1.13 nm (BCN-11), 1.05 nm (BCN-12), and 1.01 nm (bare fused silica) over a 100 $\mu m^2$ surface.

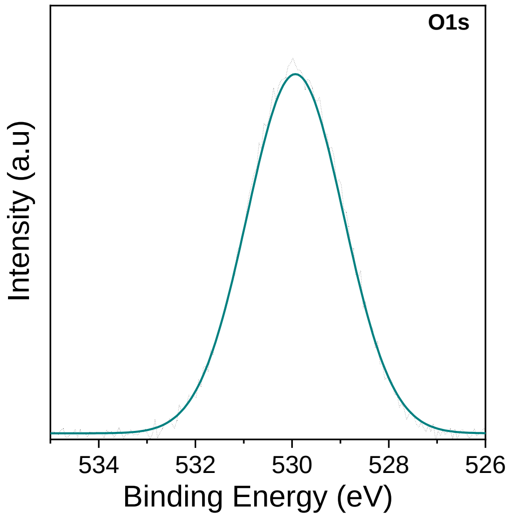


Fig S5. O1s XPS peak deconvoluted of BCN-11 (left) and BCN-12 (right).

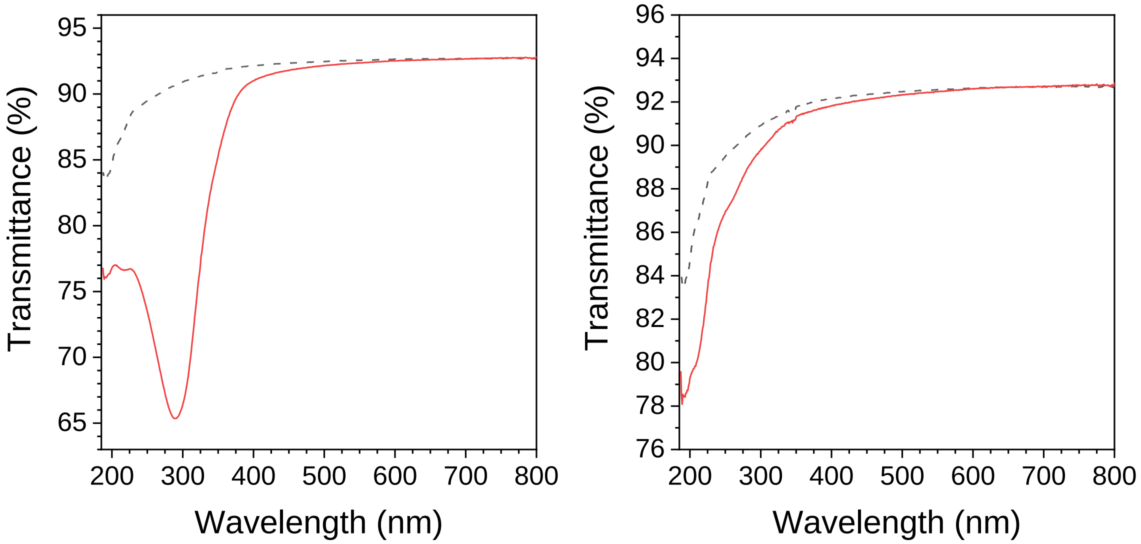


Fig S6. Transmittance of BCN-11 (left) and BCN-12 (right)

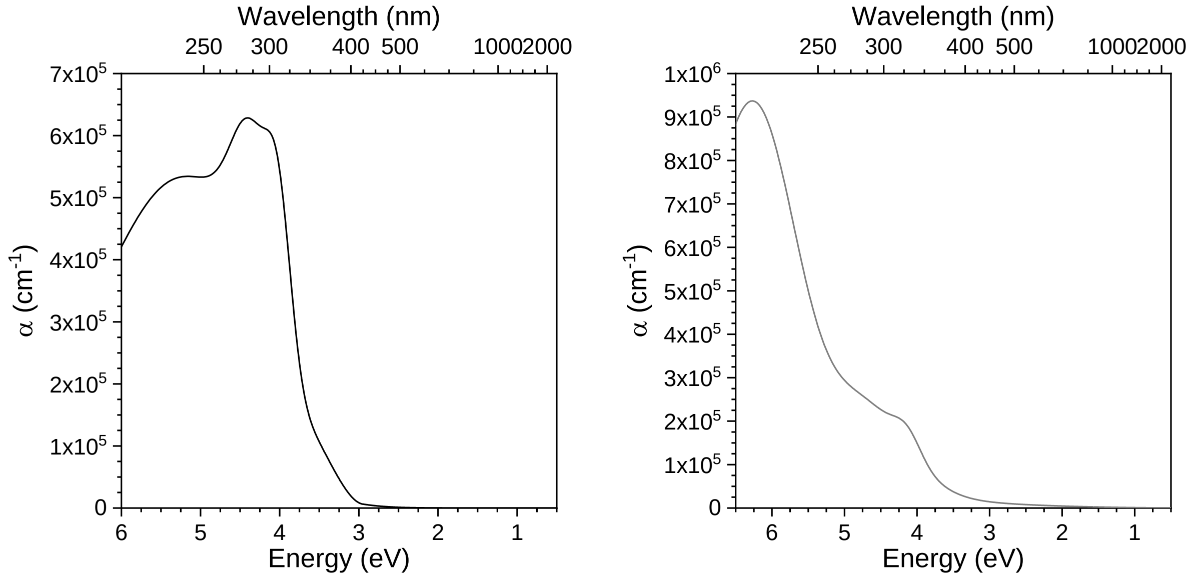


Fig S7. Absorption coefficient spectra of BCN-11 (left) and BCN-12 (right)

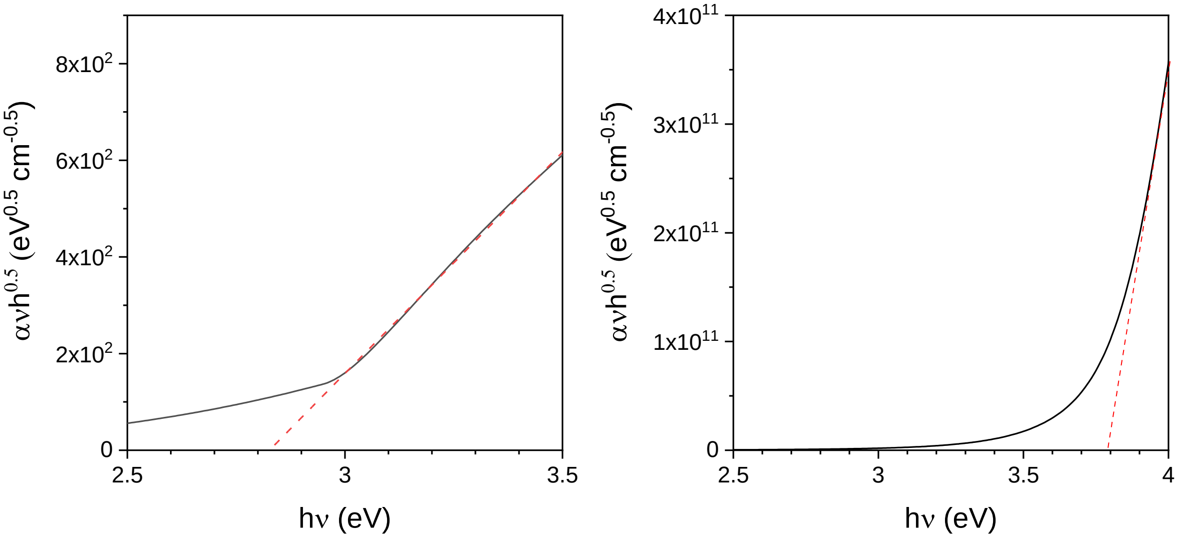


Fig S8. Tauc plots of BCN-11 (left) and BCN-12 (right)

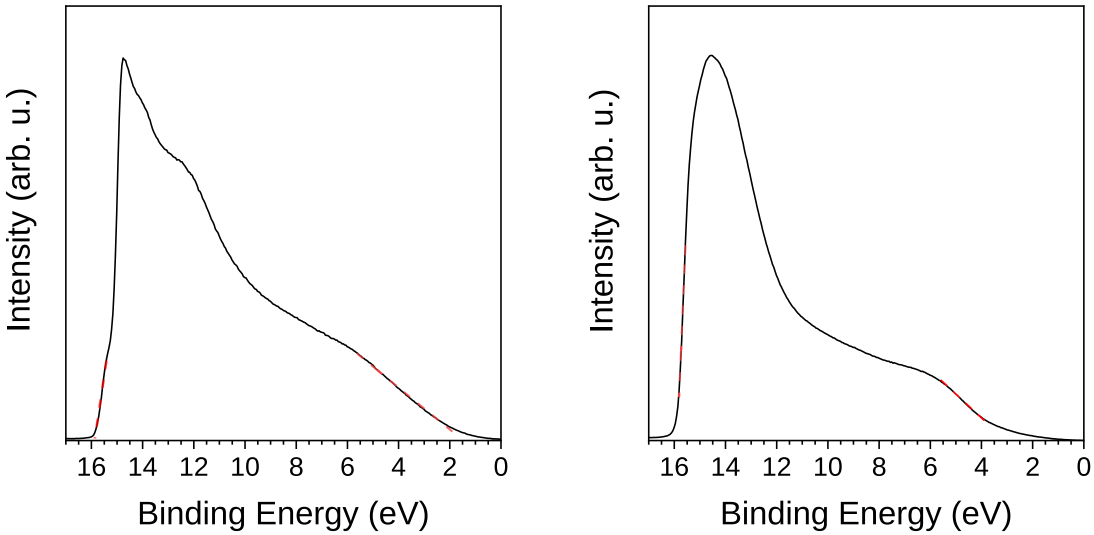


Fig S9. UPS spectra of BCN-11 (left) and BCN-12 (right).

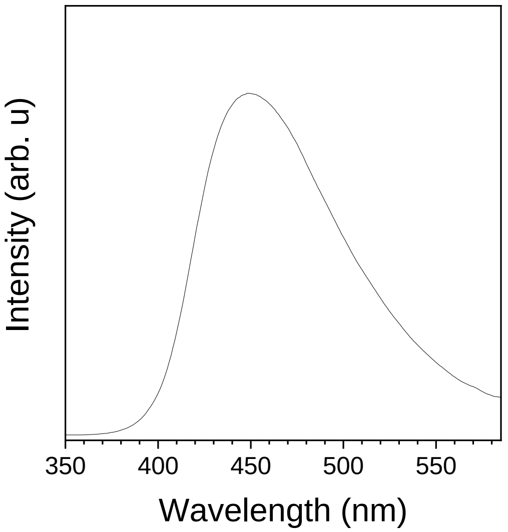


Fig S10. Fluorescence spectrum of BCN-11 thin film at 300 nm excitation.
